# Modeling Coral Reef Fish Home Range Movements in Dry Tortugas, Florida

**DOI:** 10.1155/2014/629791

**Published:** 2014-01-16

**Authors:** Nicholas A. Farmer, Jerald S. Ault

**Affiliations:** ^1^Division of Marine Biology and Fisheries, Rosenstiel School of Marine and Atmospheric Science, University of Miami, 4600 Rickenbacker Causeway, Miami, FL 33149, USA; ^2^Sustainable Fisheries Division, Southeast Regional Office, NOAA National Marine Fisheries Service, 263 13th Avenue South, St. Petersburg, FL 33701, USA

## Abstract

Underestimation of reef fish space use may result in marine reserves that are too small to effectively buffer a portion of the stock from fishing mortality. Commonly used statistical home range models, such as minimum convex polygon (MCP) or 95% kernel density (95% KD) methods, require the exclusion of individuals who move beyond the bounds of the tracking study. Spatially explicit individual-based models of fish home range movements parameterized from multiple years of acoustic tracking data were developed for three exploited coral reef fishes (red grouper *Epinephelus morio*, black grouper *Mycteroperca bonaci*, and mutton snapper *Lutjanus analis*) in Dry Tortugas, Florida. Movements were characterized as a combination of probability of movement, distance moved, and turning angle. Simulations suggested that the limited temporal and geographic scope of most movement studies may underestimate home range size, especially for fish with home range centers near the edges of the array. Simulations provided useful upper bounds for home range size (red grouper: 2.28 ± 0.81 km^2^ MCP, 3.60 ± 0.89 km^2^ KD; black grouper: 2.06 ± 0.84 km^2^ MCP, 3.93 ± 1.22 km^2^ KD; mutton snapper: 7.72 ± 2.23 km^2^ MCP, 6.16 ± 1.11 km^2^ KD). Simulations also suggested that MCP home ranges are more robust to artifacts of passive array acoustic detection patterns than 95% KD methods.

## 1. Introduction

Many terrestrial and marine vertebrates use specific areas for their movements [1, 2]. The area utilized for the majority of animal's activities and movements during certain time periods or over particular life stages is referred to as a “home range” (see review in [3]). Although numerous studies have documented home ranges for fishes (e.g., [4–9]), few have precisely quantified movements and habitat space relative to no-take marine reserve (NTMR) boundaries (but see [10]). Accurate estimates of home range size are crucial to the design of efficient NTMRs (e.g., [11–15]); an NTMR smaller than the home range size of a fish will afford it little protection from fishing pressure.

A number of statistical models have been developed to generate quantitative estimates of home range size from location data. The most commonly used methods are the minimum convex polygon method (MCP [16]), which defines the boundaries of space use and the kernel density model (KD [17]), which calculates spatial utilization probabilities. The individual's MCP or KD home range estimates generated from daily location data must reach a visible asymptote through time to be a valid estimate of home range size [16]. Within a fixed acoustic array, this assumption may create bias in resultant space-use estimates by excluding the most mobile individuals as well as individuals who are only detected at receivers placed in a straight line. Although extrapolation beyond observed values has limitations, estimating the scope of movements beyond an acoustic array in a probabilistic fashion provides a useful upper bound to empirical estimates of home range size. Underestimation of fish space requirements may lead to overconfidence regarding resource protection in the context of NTMR design [18]; therefore, a conservative upper bound is most appropriate for management use.

In this study, we developed a spatially explicit individual-based simulation model of coral reef fish movements within a home range, using simple inputs of move frequency, move distance, and move direction relative to home range center. These inputs were parameterized from acoustic telemetry data for groupers and snappers acquired from a broad-scale, long-term study of reef fish movements and habitat use in Dry Tortugas, Florida [10]. Using simple behavioral rules to replicate observed spatial detection frequency patterns, we explored potential biases and errors in commonly used home range estimation methods emerging from constraints on sample size, scope of the acoustic array, duration of tracking, and number of movements detected. We compared MCP and KD home ranges of each species derived from simulated movements to estimates of home ranges derived from observed movement data. We developed upper bounds for space use estimates for groupers and snappers, accounting for movements beyond the scope and duration of the acoustic tracking program.

## 2. Methods

### 2.1. Statistical Home Range Models

We evaluated two widely used statistical estimators of animal home range: (1) MCP and (2) KD. The basis of these methods is as follows.

The MCP is a simple heuristic “rule of thumb” model of home range. The MCP is constructed by drawing the smallest possible polygon around the observed position fixes. MCPs have been broadly used due to their computational simplicity and ease of comparison between studies, but they have been criticized for their tendency to overestimate home range size through the inclusion of unused sites [17, 19–21].

Kernel density (KD) estimation creates a histogram representation of a spatial variable (i.e., the *x* or *y* coordinate of a set of locations), constructed such that each point falls in the center of a sampling bin. In the simplest version of this method, the sampling bins overlap, and the points that are included in any bin are weighted according to a uniform distribution. Advanced KD methods improve this approach by replacing the uniform weighting function with a kernel function. This kernel function is a probability density function with a distribution defined by the following equation:

(1) $$\begin{array}{c} \hat{f}\left(x\right)=\left[\frac{1}{\left(nh^{2}\right)}\right]\sum_{i=1}^{n}K\left\{\frac{\left(x-X_{i}\right)}{h}\right\}, \end{array}$$

where *K* is the kernel that determines the shape of the distribution that is placed over each of the points; *h* is the bandwidth, which controls the search radius or width or the kernel; *n* is the number of location estimates (points) used in the analysis; and **x** and **X** refer to the vectors of the coordinates of the evaluation point and all other points, respectively.

In practical applications, a grid size is selected to represent the most favorable tradeoff between resolution (and hence smoothness of the resultant probability density function) and time (coarse grids are more quickly analyzed). For our study, a grid size of 20 m was selected. Using the Geospatial Modeling Environment [22], a Gaussian kernel function was fit to the data. Each position fix was evaluated, and each evaluation point was in turn evaluated based on all surrounding points. Points surrounded by many other points have a high density value. To determine which surrounding points would contribute to the estimation of the density at the evaluation point, a smoothing factor (bandwidth), *h*, was used to describe the search radius about the evaluation point. Most researchers do not report cell size and bandwidths used for determining home range sizes, making it impossible to compare KD home ranges between studies due to the profound influence of these parameters upon the resultant home range estimates [23]. For our study, smoothed cross-validation [24] was used to select the optimal bandwidth; this method is replicable and is believed to be robust to the overlapping position fixes commonly obtained from passive acoustic telemetry studies.

The distance from each point to the evaluation point was calculated. Based on these distances, a cumulative value was assigned to the evaluation point. This procedure was repeated until all the points in the distribution were evaluated. They were all scored and assigned density values. The values for the kernel were summed at every point on the surface. Finally, a surface was created that contained grid cell values of the kernel density estimate of the distribution. The surface was then contoured at specified volumes to give percentage home ranges (i.e., a 95% home range was contoured at 95% of the volume of the density surface—not at 95% of the area of the home range).

### 2.2. Home Range Model: Development

To evaluate the performance of various statistical estimators of coral reef fish home range, we developed a general stochastic spatially explicit individual-based movement model following the methods of Holgate [25] and Okubo [26]. In the model, the angle of rotation *θ* between the starting (**x**_0_ = (*x*_0_, *y*_0_)) and the finishing points (**x**_1_ = (*x*_1_, *y*_1_)) of movement in a given time interval Δ*t* was defined by

(2) $$\begin{array}{c} \theta =\text{ta}{\text{n}}^{-1}\left(\frac{y_{1}-y_{0}}{x_{1}-x_{0}}\right). \end{array}$$

The Euclidean distance *d* moved by a fish between **x**_0_ and **x**_1_ in the time interval is

(3) $$\begin{array}{c} d=\sqrt{{\left(x_{1}-x_{0}\right)}^{2}+{\left(y_{1}-y_{0}\right)}^{2}}. \end{array}$$

Fish home range **H** was expressed as a function of the statistical distribution of observed directions and distances of movement

(4) $$\begin{array}{c} H=\int f\left(\theta ,\overset{-}{\theta }\right)d\theta , \end{array}$$

where $f\left(\theta ,\overset{-}{\theta }\right)d\theta$ is the probability of moving in direction *θ* biased towards the direction of the individual's home range center relative to its current position, indicated by the angle $\overset{-}{\theta }$. The form of the home range function was expressed as a general von Mises distribution that approximates a normal probability density function

(5) $$\begin{array}{c} f\left(\theta ,\overset{-}{\theta }\right)=\frac{1}{\sqrt{2\pi }I_{0}\left(\kappa \right)}\exp\left[-\kappa \cos\left(\theta -\overset{-}{\theta }\right)\right] \end{array}$$

with two parameters governing the distribution of movement directions: a mean direction, $\overset{-}{\theta }$, where $-\pi \leq \overset{-}{\theta }\leq \pi$; and a concentration (variance) parameter *κ* (where *κ* ≥ 0). Higher values of *κ* are indicative of greater home range affinity. A modified Bessel function, *I*_0_(*κ*), normalized $f\left(\theta ,\overset{-}{\theta }\right)$ to integrate to 1.

The probability of moving from location **x**_0_ to **x**_1_ was expressed as probability function relating the product of the distance moved per unit time and the angle of movement:

(6) $$\begin{array}{c} K\left(x_{0},x_{1},\Delta t\right)=\frac{f\left(d\right)}{d}f\left(\theta ,\overset{-}{\theta }\right), \end{array}$$

where *K* is a kernel that projects the starting and finishing locations (**x**_0_, **x**_1_) from Cartesian to spherical coordinates, *d* is the absolute value of the displacement, and *θ* is the angle of movement between the starting and finishing points [27].

### 2.3. Home Range Model: Parameterization

**Figure 1:**
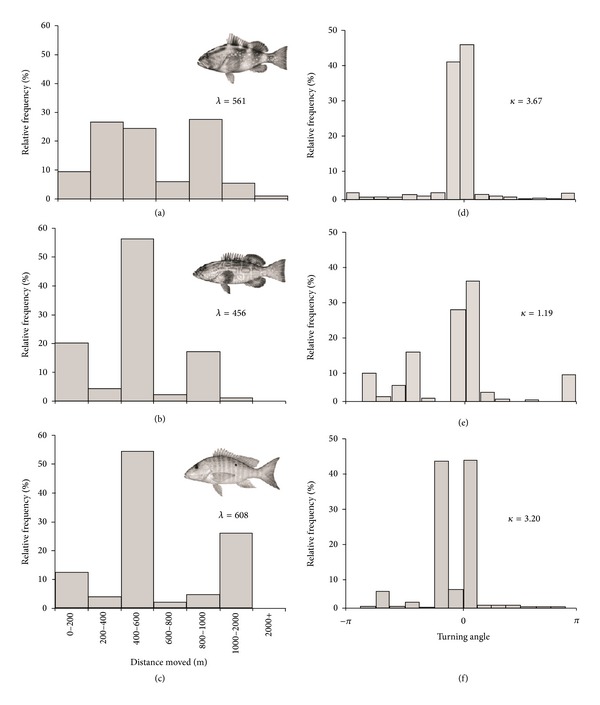
Relative frequency histograms of observed (a)–(c) nonzero movements and (d)–(f) turning angles relative to home range center for ((a), (f)) all tracked red grouper (*Epinephelus morio*), ((b), (e)) black grouper (*Mycteroperca bonaci*), and ((c), (f)) mutton snapper (*Lutjanus analis*). Means (*λ*) of exponential distribution of movement distance and concentration parameter (*κ*) of von Mises distribution of turning angles are provided on figures. Fish illustrations copyright Diane Rome Peebles.

To obtain data to parameterize the simulation model, between March 2006 and February 2008, we managed an array of 25–30 VEMCO VR2 (VEMCO Ltd., Nova Scotia, Canada; http://www.vemco.com/) hydrophone receivers in the northwestern quadrant of Dry Tortugas National Park, Florida. This array provided up to 30 km^2^ of acoustic coverage over a representative suite of depths and benthic reef habitats ([10] Figure 1). VEMCO V16 acoustic transmitters were surgically implanted in fish captured by hook and line ([10] Table 1). Unique receiver detection ranges were calibrated using methods detailed in Farmer et al. [28].

Over two million acoustic detections were registered for several species of tagged coral reef fish (Table 1). Red grouper, *Epinephelus morio *(*n* = 45), were the primary emphasis for this study, with sensitivity runs performed using parameterizations from the movements of opportunistically tagged black grouper, *Mycteroperca bonaci *(*n* = 3), and mutton snapper, *Lutjanus analis *(*n* = 3). To mitigate the potential impacts of transmitter signal collisions, tags were set with random delay times of 30–180 sec, and fewer than 5 fish were tagged at any given location. Model-weighted mean position estimates generated on 5-min batching intervals were considered optimal, following Farmer and Ault [10] and Farmer et al. [28].

For our home range model input parameters to be representative of population trends, it was important that individual fish were not overrepresented. Theoretically, risk of overrepresentation in the input parameters for the model would be higher for fish with longer tracking durations or higher total detections. To quantitatively identify minimal standards for tracking duration or number of detections and to evaluate the influence of individual fish (Table 1) on input parameters, the relationships between red grouper estimated home ranges and number of detections, number of detected moves and tracking duration were evaluated using linear regression models [29], with fish ID as a random factor. Additional linear regressions were used to test for relationships between days tracked versus number of detected moves, and total detections versus number of detected moves. A significant relationship between any of these combinations of variables would suggest that parameterization of home range models might be overly influenced by a limited subset of actively moving fish.

A custom Java 6.10 algorithm was developed to process time-sequenced observed reef fish movements paths (“observed”) to estimate distances from previous positions, *d*_prev_; distances from home range center, *d*_centroid_; and turning angles, *θ*, between movements, relative to angle to home range centroid, $\overset{-}{\theta }$. Data were pooled within species. Movements were expressed as a three-step process: (1) a probability of moving during the interval, (2) a distance moved, and (3) a direction moved (if movement occurred). Movement distances were modeled using “Input Analyzer for Arena” (Rockwell Automation, Warrendale, PA) to determine the best fit probability distribution for *d*_prev_. Species-specific aggregated turning angles relative to home range center ($\theta -\overset{-}{\theta }$) were fit to von Mises distributions with the “Circular” package in R (http://www.r-project.org/).

The sensitivity of the model parameterization to lower sample sizes was evaluated in two ways. First, to test the impacts of only low sample size, 1000 bootstrapped samples of 437 randomly selected movement observations from all red grouper movement observations (*n* = 16, 821) were fit to exponential distributions, and the mean was compared to the mean for an exponential distribution fit to all red grouper observations. This threshold was selected because only 437 movements were observed from two black grouper. Because meaningful movement data were only obtained from two black grouper and two mutton snapper, the impacts of a limited number of individuals upon model parameterization was also tested. To test the impacts of having movement observations from a limited number of individuals, two observed red grouper were randomly selected, and 500 bootstrapped samples of 437 randomly selected observed movements were fit to exponential distributions. This test was iterated 100 times, and the mean across iterations was compared to the mean for an exponential distribution fit to all red grouper observations.

### 2.4. Home Range Model: Simulation Runs

Simulated movement paths and detection sequences were generated using a spatially explicit model implemented in Java 6.10 using the Repast Agent-based Modeling Toolkit [30, 31]. This model parsed the Dry Tortugas, Florida, region into 22,620 cells of 200 m by 200 m size. Each cell was assigned a unique identification code and depth. An array of simulated receivers was implemented in grid cells corresponding to the locations of receivers described in Farmer and Ault [10]. For each simulation run, fish were instantiated with home ranges centered atop each of the simulated receivers.

For simulations, if a random number drawn from a uniform distribution between 0 and 1 was less than the probability of moving a discernible distance (*ψ*) in a 5 min interval, a movement distance was randomly drawn from the exponential distribution with mean (*λ*) fit to *d*_prev_. Maximum movement distance was bounded at 6000 m per move, as sustained swimming speeds above 72 km·h^−1^ (45 mph) were deemed unrealistic for reef fish. Movement distances were converted from meters to simulation grid cell units (i.e., 200 m units). Movements of <200 m (i.e., grid cell distance <1 unit) were beneath the spatial resolution of the position estimates emerging from the telemetry data [28] and were considered negligible. Turning angles relative to home range center were randomly selected from a von Mises distribution with the concentration parameter (*κ*_*τ*_) for the species under examination.

A simulated array of receivers was used to provide more accurate comparisons between empirical and simulated data. The number of detections registered by a simulated receiver during a given interval for each fish was computed by dividing the length of the interval by the ping rate and then multiplying by the probability of detection at distance [28]. Simulated movements (“Sim-Actual”) and detection patterns (“Sim-Detect”) were recorded for one year.

### 2.5. Home Range Models: Evaluation

Observed and simulated detections were postprocessed using a model-weighted harmonic mean positioning estimator [10, 28] which increased positioning resolution during intervals when detections were recorded at multiple receivers. Home ranges of observed and simulated fish were compared. MCP and 95% KD home ranges were computed for “Observed,” “Sim-Detect,” and “Sim-Actual” position fixes using Geospatial Modeling Environment [22]. KD home ranges were computed using smoothed cross-validation (SCV) bandwidth estimation and a cell size of 20 m. The spatial distribution of error introduced into empirical studies by fish movements beyond the bounds of the array was evaluated by comparing “Sim-Detect” and “Sim-Actual” home ranges for fish with home range centers at core and peripheral receivers. Receivers were defined as “peripheral” if they were not completely surrounded by other receivers.

Although it is broadly accepted that an empirical home range estimate should only be considered reliable if an asymptote is present when estimated home range size is plotted against tracking duration, little guidance exists in the literature regarding minimum tracking duration or detections, what percent change in home range size constitutes a “useful” asymptote, or how to account for likelihood of movements beyond the study area (e.g., “peripheral effects”). To explore the sensitivity of empirical home range estimates to these criteria, we computed MCP and KD home ranges for observed fish under a variety of filters. We also developed a generalized linear mixed model using SAS Proc GLIMMIX (SAS Institute, Inc., Cary, NC) to test the relationship between percentage of final estimated red grouper home range size versus the fixed effect of tracking duration (months) and random effects of individual and interaction effects. Additionally, estimated home range size for simulated fish was plotted against months of simulated observation to examine asymptotic characteristics.

## 3. Results

### 3.1. Home Range Model: Parameterization

Input data did not appear significantly biased towards overestimation of movement due to an overabundance of sampling points from fish with large home ranges. No trends were detected between observed red grouper home ranges and number of detections or moves (*P* > 0.05), days tracked and number of moves detected (*F*_1,42_ = 0.79, *P* > 0.05), or total detections and number of moves detected (*F*_1,42_ = 1.02, *P* > 0.05). These results suggest that simulation model input parameters were relatively insensitive to the length of observation. By contrast, a slightly declining trend between days tracked and observed red grouper home range size (*β* = −0.01, *F*_1,42_ = 9.75, *P* < 0.01) was detected, suggesting that fish leaving the acoustic array shortly after tagging may have been transient fish, may have been transitioning between different home ranges, or may have been behaviorally impacted by the tagging process. Regression models failed to identify quantitative thresholds for tracking duration, number of detections, or number of moves for estimating a home range. However, when the analysis of home range size versus days tracked was restricted to observed red grouper exhibiting an asymptotic home range size estimate and with a home range center towards the middle of the acoustic array (i.e., “low peripheral effects”), the relationship between days tracked and home range size was eliminated (*F*_1,11_ = 2.10, *P* > 0.05). This finding implies that home range estimates of these individuals was robust to sampling duration but may also suggest that the common practice of restricting home range estimates to fish meeting the asymptotic assumption may underestimate mean space requirements by excluding more mobile individuals from consideration.

Input parameters for the home range model are presented in Table 1. Empirical observations of reef fish movements indicated that red grouper and black grouper moved infrequently (<1% of 5 min intervals) as compared to mutton snapper (5.76%). For all three species, exponential distributions provided the best fits with the lowest mean square error to the nonzero movement data (Table 1, Figure 1); however, some multimodality was evident in the distribution of distances moved, resulting in relatively poor fits (*P* < 0.05). The best fit von Mises distributions for turning angles are displayed in Figure 1. The von Mises concentration parameter was three times higher for red grouper versus black grouper, suggesting a comparatively stronger association with a home range center for red grouper.

**Figure 2:**
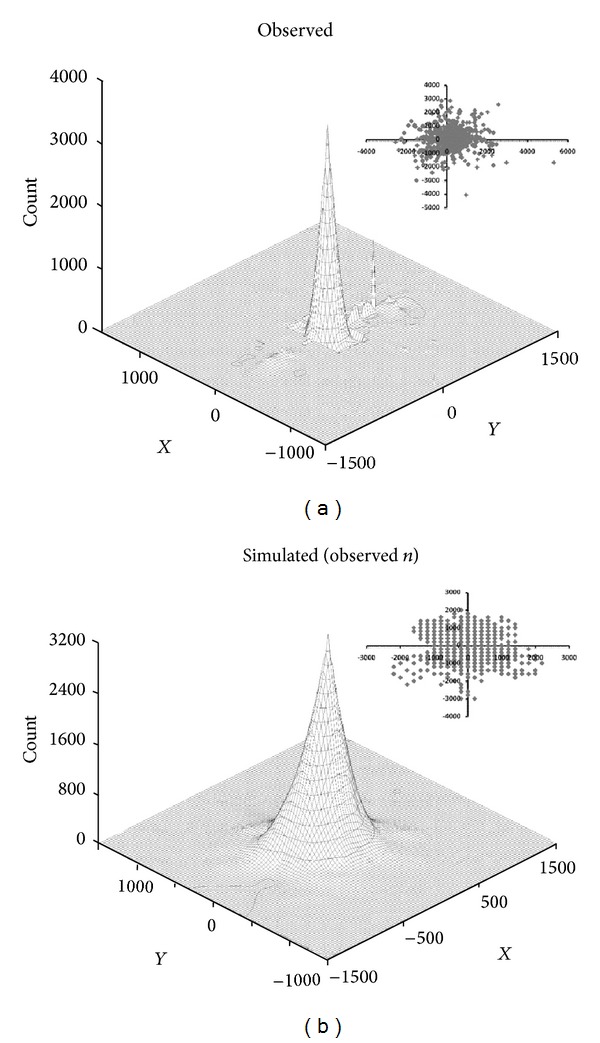
Distribution of spatial positions relative to home range center (m) for (a) observed and (b) simulated red grouper, aggregated across all individuals. Insets show two-dimensional distribution of position fixes. Simulation output is shown for movements corresponding to empirical sample size.

The observed spatial distribution of movements relative to home range centers was reasonably captured by the simulation (Figure 2); however the emergent pattern from the “Sim-Detect” movements was more circular and uniform than the real-world pattern. The shape of the distribution was controlled by the interplay of the two movement parameters (*λ*, *κ*_*τ*_); the magnitude of the distribution was controlled by the frequency of movement (*ψ*) and the number of individuals tracked. For black grouper and mutton snapper, the shapes of the simulated distributions were very similar to those observed. For red grouper, the shape of the observed distribution was more heavily concentrated on the home range center than that of the simulated distribution. The largest movement observed for red grouper was slightly less than 600 m.

Small sample sizes appeared to have little impact on movement model fits, provided that they were randomly sampled and representative of the population; there was no significant difference (*P* > 0.05) between the exponential distribution fit mean (*λ*) based on 16,821 red grouper movement observations (561.4 ± 2.8) and the exponential distribution fit *λ* based on 1000 bootstrapped samples of 437 randomly selected red grouper observations (560.6 ± 0.55). However, if movements were not representative of the population, model parameterization could be heavily skewed; there was a substantial difference between the exponential distribution fit *λ* based on 500 bootstrapped samples of 437 randomly selected red grouper observations drawn from 100 randomly selected pairs of observed red grouper (747.15 ± 34.4; range 288.4–2260.3) and the exponential distribution fit mean (*λ*) based on all 16,821 red grouper observed movements (561.4 ± 2.8). These findings suggest that the relatively low number of observed movements for the two observed black grouper and two observed mutton snapper may provide reasonable movement model fits, but only if the distributions of movement distances for those individuals were representative of the population. As this assumption was unlikely to be met, subsequent statistics for mutton snapper and black grouper are provided for contrast to red grouper only and should not be interpreted as representative of a population mean.

### 3.2. Home Range Models: Evaluation

Mean MCP and 95% KD home range estimates for “Observed,” “Sim-Detect,” and “Sim-Actual” fish positions are shown in Table 2. KD home range estimates could not be generated for some fish due to an overabundance of detections at 1-2 sites impairing SCV bandwidth estimator performance. MCP and 95% KD home range estimates differed substantially within species. MCP home range estimates for mutton snapper were larger than those of red grouper and black grouper for all three groups. Surprisingly, red grouper observed 95% KD home ranges were larger than those of mutton snapper or black grouper. By contrast, mutton snapper had the largest “Sim-Actual” and “Sim-Detect” 95% KD home ranges. MCP home range estimates appeared to be more robust to the detection limitations of the array, as there was substantially less deviation between MCP home range estimates across the three groups for each of the species. The 95% KD estimates for the “Sim-Detect” group were overinflated relative to the “Sim-Actual” estimate; this may indicate the undesirable influence of repeated observations at the same site, as well as reduced position estimates at interreceiver locations. By contrast, MCP home ranges for the “Sim-Detect” group were underestimated due to the lack of peripheral detections. The distinction between the MCP and 95% KD home range estimation methods was most pronounced for mutton snapper, the most mobile species evaluated. Long distance movements generated a large MCP for this species, but the 95% KD for observed mutton snapper was smaller than that for observed red grouper. Interestingly, 95% KD estimates for the “Sim-Actual” group were larger than MCP estimates for red grouper and black grouper.

Paired *t*-tests for means between “Sim-Actual” and “Sim-Detect” MCPs indicated that 12-month home ranges were consistently underestimated by the simulated acoustic array for black grouper (*t* = −2.43, df = 30, and *P* < 0.05) and mutton snapper (*t* = −6.37, df = 30, and *P* < 0.001) but not red grouper (*t* = −1.24, df = 30, and *P* > 0.05). This suggests that acoustic array configuration was adequate for red grouper home range estimation. A comparison of 100% MCP and 95% KD home range areas for “Sim-Detect” versus “Sim-Actual” paths showed relatively minor differences for red grouper and black grouper; however, mutton snapper “Sim-Actual” MCPs were substantially larger than those computed from simulated detections, suggesting frequent, broad-scale movements out of the simulated array.

**Figure 3:**
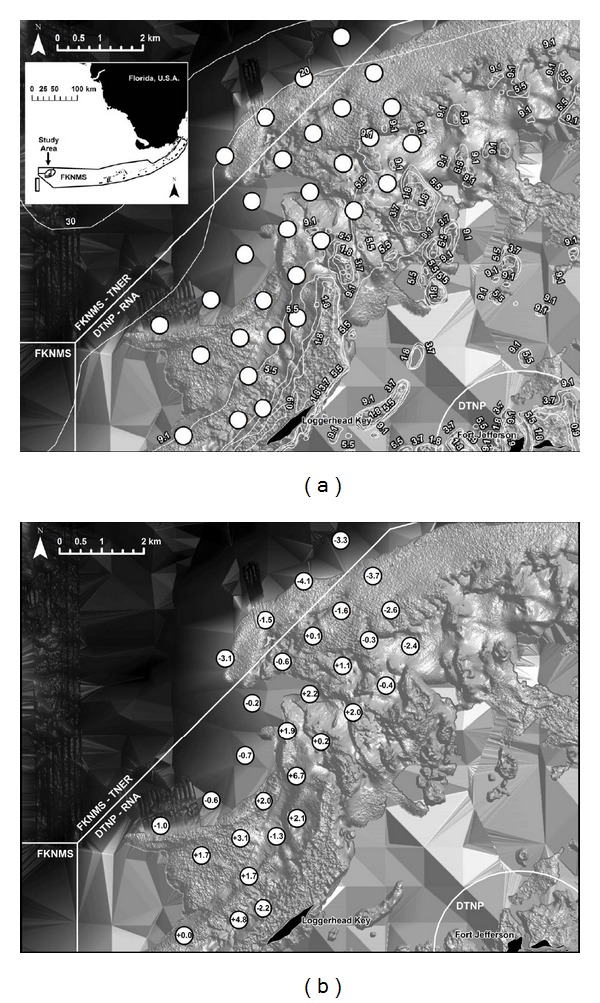
(a) Configuration of acoustic array relative to bathymetry (m) and management boundaries and (b) systematic bias in estimated red grouper home range size attributable to location of individual home range centers. White circles indicate locations of simulated acoustic receivers; numbers within circles indicate percent of under- or overestimation by minimum convex polygon (MCP) home range method for simulated fish with home range center at location of circle. Inset in (a) shows study area relative to state and federal management boundaries. FKNMS: Florida Keys National Marine Sanctuary; DTNP: Dry Tortugas National Park; RNA: Research Natural Area; TNER: Tortugas North Ecological Reserve.

**Figure 4:**
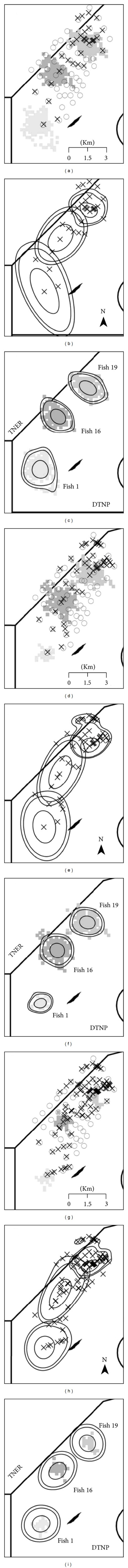
Configuration of simulated acoustic array (white circles) relative to actual path (gray squares) and simulated detections (×), with 95%, 90%, and 50% kernel density home ranges for simulated detections and actual simulation path of red grouper (a)–(c), black grouper (d)–(f), and mutton snapper (g)–(i).

Location of the home range center with respect to the configuration of the acoustic array influenced “Sim-Detect” home range size relative to “Sim-Actual” home ranges. Simulated receivers tended to overestimate home ranges for fish with home range centers in the core of the array and underestimate home ranges for fish with home range centers on the periphery of the array (Figure 3). One-tailed two-sample *t*-tests assuming equal variances revealed significantly underestimated home range sizes for red grouper (*t* = −2.41, df = 29, and *P* < 0.001), black grouper (*t* = −3.82, df = 29, and *P* < 0.001), and mutton snapper (*t* = −1.96, df = 29, and *P* < 0.05) with home range centers along the edges of the acoustic array versus those with home range centers in the core of the array. The configuration of the acoustic array may also distort the shapes and sizes of KD home ranges; comparisons between “Sim-Detect” and “Sim-Actual” KD home ranges showed “Sim-Detect” KD home ranges to be linearly distorted corresponding to receiver locations and overextended into the periphery relative to analogous “Sim-Actual” KD home ranges (Figure 4).

**Figure 5:**
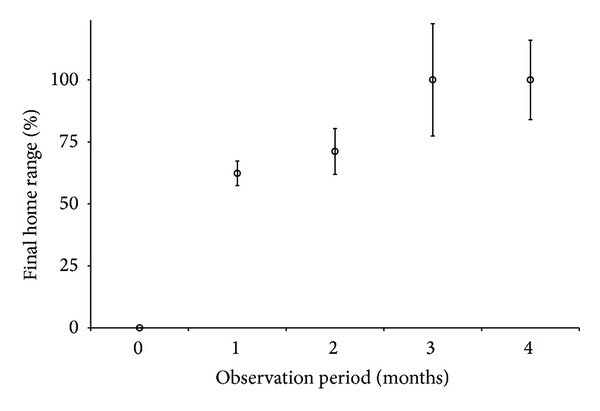
Least squares mean estimates from mixed model regression of percent of final minimum convex polygon (MCP) home range attained versus months of observation for red grouper. Bars denote standard error.

Home range estimates for individual observed tagged fish are presented in Table 3. Various filtering criteria were applied to home range estimates to explore sensitivity of species-specific home range estimates (Table 4). The most conservative filter (Table 4: bold) resulted in substantial increases in estimated home range for black grouper, no estimate for mutton snapper, and minimal changes for red grouper despite cutting sample size by over 50%. This analysis suggests that a large sample size (i.e., many tagged individuals) and a conservative filter likely provide the most robust home range estimates for fish remaining within the acoustic array. Tracking duration was a significant factor in estimated MCP home range size. Mean tracking duration from Farmer and Ault [10] was approximately three months. Least squares mean estimates generated from a mixed model regression of the percent of final MCP home range size versus months of tracking indicated that the most observed red grouper would reach nearly 100% of their final estimated home range size after 2-3 months of observation (Figure 5).

## 4. Discussion

Home range modeling approaches are generally of three types [32, 33]: (1) analytical modeling derived from statistical physics [27, 34, 35], (2) individual-based modeling developed based on optimal foraging theory [36], and (3) statistical modeling based on life history dynamics and behavioral ecology [2, 17]. In this study, we employed a hybrid statistical-analytic modeling process in an individual-based simulation environment to explore the sensitivity of commonly used statistical home range models to acoustic array design, tracking duration, and interspecific differences in fish home range movements. Our simulation model replicated basic features of observed reef fish space utilization patterns and was useful in identification of potential biases in home range estimates.

We generated MCP and KD home range estimates from empirical observations and simulation model outputs. Diagnostic testing suggested that statistical home range models were most robust for red grouper. This is not surprising, as field observation sample size for this stock was much higher than sample sizes for black grouper or mutton snapper. Bootstrapped simulations suggested that model parameterization was robust to small sample size, provided that sampled fish movements were representative of the population; thus, the results for black grouper and mutton snapper may be useful for the contrast they provide with red grouper and possibly also to understand some unique characteristics of the space requirements for these species.

Our home range simulation model parameters had simple biological and physical interpretations. When considered jointly with probability of movement (Ψ), the mean of the exponential distribution for movement (*λ*) is a proxy for overall mobility; a large *λ* value indicates that the species is capable of moving substantial distances. Similarly, the concentration parameter *κ* indicates affinity to a home range center; larger values of *κ* imply higher affinities to a core home range. Red grouper affinity to a home range center was nearly three times that for black grouper. Simulations suggested that red grouper and black grouper have similar-sized home ranges, but black grouper had a limited range and were less associated with core habitats. On the other hand, red grouper made periodic forays to nearby sites but often returned to a core habitat. Both groupers are ambush predators; however, black grouper are more piscivorous and less associated with the bottom [37, 38]. Mutton snapper exhibited higher mobility than groupers but maintained a strong association within a home range center. Mutton snapper in the Dry Tortugas are usually observed in low relief hard bottom habitats, patrolling sand channels along reef edges.

Comparing empirical animal location data with predictions from mechanistic or individual-based models is challenging [39], requiring an appropriate choice of sampling interval for discretizing continuous paths [34, 40]. We simplified this decision by creating a simulated array of acoustic receivers to replicate field detection patterns. As expected, simulated movement patterns were more homogeneous than empirically observed patterns. The duration and consistency of tracking across all individuals provided smoothing that may have masked individual patterns. In addition, many aspects of population dynamics, demographics, and life history influence fish movement patterns. Many of these factors were not captured by this simple modeling approach. For example, fish communities are comprised of both mobile and sedentary fractions [41], although it is unclear whether populations or individuals only exhibit one strategy or switch off occasionally throughout their lives [42]. Our model is most appropriate for describing the routine movements of an organism within a home range.

The multimodality observed in the input distributions of distances moved (Figures 1(a)–1(c)) may be attributable to occasional large movements such as those associated with spawning migrations, low sample sizes for black grouper and mutton snapper, and the inclusion of individuals that did not appear to utilize a home range. Additional behavioral rules could be added to our model to capture biphasic reef fish movement strategies, such as ontogenetic migrations [43] or directed spawning migrations [10, 44, 45]. Alternatively, drawing movements from the observed distribution of movements rather than a smoothed statistical distribution might also provide greater heterogeneity in simulation output. The individual-based nature of the model design simplifies incorporation of these terms or other behaviors such as territoriality, visitation of foraging or cleaning sites, or avoidance of particular habitats. Although our data provided some hints that these factors might be important, the accurate parameterization of these additional behavioral terms would be challenging.

In general, simulation data indicated that home range estimates for observed red grouper were robust to movements beyond the array within the tracking duration because sample size was high enough to allow computations to be restricted to only include fish whose movements were constrained by the array. Additional analyses of empirical data failed to identify a relationship between estimated home range size from field observations and tracking duration. Analyses suggested that 3-4 months were a reasonable time period to obtain a reliable home range estimate for red grouper. Estimates of home range size for observed fish presented in Table 2 were constrained by the assumptions of statistical home range methods, namely, that home range estimates must reach an asymptote when plotted over time as area-observation curves [46]. This led to the exclusion of 1 of 3 black grouper (33%), 12 of 45 red grouper (27%), and 1 of 2 mutton snapper (50%). Most of these organisms were excluded because they moved outside the acoustic array. As such, their actual home ranges may have been larger than the individuals whose home ranges were estimated or these animals may have relocated their home ranges. Because our simulations incorporated movements and turning angles from all individuals tracked within each species, it is not surprising that simulated reef fish movements resulted in larger estimates of home range size.

Empirical observations suggested that exploited groupers and snappers require at least 1-2 months of monitoring before their space use reaches a visually recognizable asymptote [10], and 3-4 months is an optimum monitoring period. Estimated space use in a passive tracking study is a stepwise process, with substantial leaps in cumulative home range estimates each time a new receiver registers detections. The likelihood of detections at peripheral sites increased with time, tighter spacing of the array, and/or increased mobility of the fish. Our simulations suggested that researchers seeking to empirically estimate home range size should tag fish near the core of their acoustic array, attempt to track them for at least several months, and seriously consider the tradeoffs between desired positioning resolution and the risk of undetected movements outside the array ([28], this study). Unfortunately, most reef fish movement studies to date have been limited in both scope and scale (review in [10]). Researchers should also be aware that 100% MCP and 95% KD home range estimates can be substantially different, even when generated from the same data ([47, 48], this study). Our simulation indicates that the MCP method is more robust than the 95% KD with SCV bandwidth estimation to artifacts of acoustic tracking programs utilizing a passive array, such as high numbers of repeated positioning estimates from the same location. The common lament that MCP methods may overestimate home range size [17, 19–21] was not borne out in our study. The large 95% KD estimates further stress the need for researchers to report home ranges using both methods and reporting the bandwidth estimation method and cell size for their KD estimation procedure, as these factors may have substantial impacts on resultant home range estimates.

Understanding the daily space requirements of exploited stocks is critical to the efficient design of no-take marine reserves (NTMRs). The protection afforded to a stock by an NTMR is dependent upon the fishing pressure surrounding the NTMR and the percentage of time the “protected” portion of the stock spends outside NTMR boundaries. Most models of marine protected areas either assume no fish movement or grossly oversimplify the dynamics of fish mobility [49–59]. The simple modeling process employed in this study was based on the concept that mobility of an organism is a process of three linked behaviors: probability of movement, distance moved, and turning angle. An organism that moves frequently (e.g., high *ψ*) or moves large distances (e.g., high *λ*) may not be “highly-mobile” in the strictest sense if it has a high affinity to a home range center (e.g., high *κ*) that restricts its overall dispersal; however, the percentage of time spent beyond the bounds of the protected area becomes a critical consideration in NTMR planning. The simple formulation of our model allows for easy manipulation of home range affinity and distances moved, allowing for easy extension to various movement strategies and extrapolation to new species as data become available. Our model provided useful upper bounds for empirically observed home range estimates. It could be easily extended into spatial population dynamic simulation models to assess NTMR performance or suggest appropriate NTMR designs relative to the spatial scale of movements.

## Figures and Tables

**Table 1:** Input parameters for individual-based mechanistic localizing tendency movement model, based on observed movements of reef fish in Dry Tortugas, Florida.

Common name	*n*	Detections	Moves	*ψ*	*λ* ± SE	*κ* ± SE
Red grouper	44	2,103,428	16,821	0.80%	561 ± 3	3.67 ± 0.04
Black grouper	2	84,108	437	0.56%	456 ± 14	1.19 ± 0.09
Mutton snapper	2	22,006	1,267	5.76%	608 ± 11	3.20 ± 0.11

Note: *n* denotes number of fish with detected movement used to parameterize model, *ψ* denotes probability of movement during 5 min interval, *λ* denotes mean of exponential distribution of movement distances, and *κ* denotes mean of von Mises distribution of turning angles between movements, relative to home range center.

**Table 2:** Estimated home range areas (km^2^) for empirically observed red grouper, black grouper, and mutton snapper compared to home range areas from simulated detection patterns and actual paths for simulated fish, with difference between simulated and simulated detection home range sizes.

Species	Stat	Minimum convex polygon (MCP), home range (km^2^)				Kernel density (95% KD), home range (km^2^)			
		Observed	Simulated	Simulated detections	Simulated-simulated detections	Observed	Simulated	Simulated detections	Simulated-simulated detections
Red grouper	Mean	2.09	2.28	2.49	−0.19	1.71	3.60	7.72	−4.12
	sd	2.04	0.81	1.58	1.85	0.48	0.89	4.69	4.75
	*n *	29	32	30	30	29	32	29	29
	se	0.38	0.14	0.29	0.34	0.09	0.16	0.87	0.88

Black grouper	Mean	1.44	2.06	1.84	0.34	0.24	3.93	7.16	−3.42
	sd	1.48	0.84	1.33	1.65	0.12	1.22	4.70	4.79
	*n *	2	32	30	32	2	32	29	29
	se	1.05	0.15	0.24	0.29	0.08	0.22	0.87	0.89

Mutton snapper	Mean	7.64	7.72	5.55	2.17	0.58	6.16	8.01	−1.85
	sd	—	2.23	3.06	3.67	—	1.11	3.80	4.14
	*n *	1	32	32	32	1	32	32	32
	se	—	0.39	0.54	0.65	—	0.20	0.67	0.73

**Table 3:** Empirical movement and home range (MCP: minimum convex polygon; KDE: kernel density estimate; km^2^) estimates from acoustically tracked red grouper (RG), black grouper (BG), and mutton snapper (MS) in Dry Tortugas, Florida.

Species	ID	Total length (cm)	Peripheral effect	Days tracked	Asymptote	Home range (km^2^)		Detections	% total	Moves	% total
						MCP	95% KD				
RG	36	64	Moderate	18	No	8.16	12.21	2,198	0.1%	300	1.8%
RG	37	47	Low	179	Yes	0.47	0.10	64,672	3.1%	85	0.5%
RG	41	47	Low	179	Yes	0.44	0.09	75,732	3.6%	61	0.4%
RG	42	48	Low	179	Yes	0.93	0.07	21,823	1.1%	484	2.9%
RG	47	48	Moderate	11	No	12.3	17.16	1,534	0.1%	57	0.3%
RG	51	66	Low	157	Yes	1.95	0.17	12,329	0.6%	1149	6.8%
RG	170	61	High	93	No	Linear	Linear	705	0.0%	11	0.1%
RG	171	60	High	98	Yes	0.64	4.89	2,359	0.1%	23	0.1%
RG	172	49	High	3	No	5.14	7.39	471	0.0%	29	0.2%
RG	173	49	Moderate	92	Yes	1.28	4.44	611	0.0%	116	0.7%
RG	175	53	Moderate	96	No	Linear	Linear	5,190	0.3%	8	0.0%
RG	176	55	High	93	Yes	0.64	3.21	4,512	0.2%	32	0.2%
RG	177	50	High	89	No	Linear	Linear	373	0.0%	6	0.0%
RG	178	65	High	90	No	Linear	Linear	2,591	0.1%	193	1.1%
RG	179	57	Moderate	23	Maybe	7.39	19.90	244	0.0%	36	0.2%
RG	180	55	Moderate	40	Maybe	3.19	12.17	380	0.0%	93	0.6%
RG	181	49	High	13	Maybe	1.97	4.07	883	0.0%	38	0.2%
RG	183	48	High	98	Yes	3.22	0.01	8,491	0.4%	1325	7.9%
RG	184	55	Low	43	Yes	1.91	1.06	4,915	0.2%	848	5.0%
RG	185	55	Low	43	Yes	1.9	0.66	8,895	0.4%	171	1.0%
RG	186	51	Moderate	91	Yes	3.49	0.55	12,327	0.6%	1259	7.5%
RG	187	50	Low	92	Yes	1.89	1.31	4,806	0.2%	965	5.7%
RG	190	62	High	101	Yes	Linear	Linear	55,093	2.7%	25	0.1%
RG	191	51	High	93	Yes	0.63	0.85	36,329	1.8%	38	0.2%
RG	194	54	High	94	Maybe	1.55	0.88	12,928	0.6%	106	0.6%
RG	862	54	Very high	86	Maybe	0.92	1.00	73,728	3.6%	26	0.2%
RG	863	51	High	95	Yes	0.31	5.32	6,728	0.3%	7	0.0%
RG	864	55	High	92	No	Linear	Linear	44,890	2.2%	892	5.3%
RG	865	56	High	33	Yes	1.28	0.39	14,616	0.7%	1027	6.1%
RG	866	53	High	66	No	Linear	Linear	424	0.0%	73	0.4%
RG	867	55	High	89	Yes	0.49	0.12	42,058	2.0%	2687	16.0%
RG	868	49	High	88	No	Linear	Linear	24,533	1.2%	489	2.9%
RG	869	60	High	87	No	Linear	Linear	21,703	1.0%	2978	17.7%
RG	870	45	High	25	Yes	2.2	2.25	2,528	0.1%	354	2.1%
RG	871	57	High	76	No	Linear	Linear	400	0.0%	103	0.6%
RG	872	53	High	79	Yes	0.49	0.19	70,316	3.4%	77	0.5%
RG	873	48	Low	280	Yes	6.44	0.01	199,933	9.6%	5	0.0%
RG	874	60	Low	278	Yes	1.4	0.12	313,240	15.0%	173	1.0%
RG	875	52	Low	280	Yes	1.68	0.69	205,442	9.9%	29	0.2%
RG	877	57	Low	280	Yes	0.83	0.33	124,104	6.0%	93	0.6%
RG	878	48	Moderate	44	Yes	5.76	2.96	21,144	1.0%	201	1.2%
RG	880	49	Low	269	Yes	2.59	4.38	35,437	1.7%	14	0.1%
RG	881	50	Low	280	Yes	9.17	1.64	261,025	12.6%	27	0.2%
RG	884	53	Low	280	Yes	1.68	0.25	272,798	13.1%	108	0.6%

BG	43	75	Low	179	Yes	2.48	0.12	72,644	86.4%	410	93.8%
BG	174	50	High	91	Yes	0.39	0.35	11,466	13.6%	27	6.2%

MS	50	43	High	4	No	0.19	0.23	183	0.8%	39	3.1%
MS	53	70	Moderate	168	Yes	7.64	0.58	21,825	99.2%	1228	96.9%

Note: KDE bandwidths (*x, y,* and* xy covariance*) available upon request.

**Table 4:** Availability and sensitivity of minimum convex polygon (MCP) and 95% kernel density (KD) home range (km^2^) estimates from acoustically tracked reef fish in Dry Tortugas, Florida (from [10]) to filtering criteria.

Species	Days	Detections	Asymptote	Peripheral effect	*N*	MCP		95% KD	
						Mean	SE	Mean	SE
Red grouper	All	All	All	All	44	2.77	0.44	3.26	0.75
Red grouper	All	All	All	Low to moderate	22	3.56	0.71	3.82	1.29
Red grouper	All	All	All	Low	14	2.38	0.65	0.78	0.31
Red grouper	>30	All	All	All	38	2.04	0.33	1.71	0.43
Red grouper	>60	All	All	All	33	1.88	0.37	1.33	0.31
Red grouper	All	All	Yes or maybe	All	32	2.22	0.38	2.39	0.73
Red grouper	All	All	Yes	All	27	2.07	0.41	1.39	0.33
Red grouper	All	>1000	All	All	35	2.60	0.50	2.17	0.65
**Red grouper**	**>60**	**>1000**	**Yes**	**Low**	**12**	**2.46**	**0.76**	**0.73**	**0.36**
*Red grouper *	*>30 *	*All *	*Yes or maybe *	*Low to high *	*28 *	*2.08 *	*0.39 *	*1.74 *	*0.51 *

Black grouper	All	All	All	All	2	1.44	1.05	0.24	0.12
Black grouper	All	All	All	Low to moderate	1	2.48		0.12	
Black grouper	All	All	All	Low	1	2.48		0.12	
Black grouper	>30	All	All	All	2	1.44	1.05	0.24	0.12
Black grouper	>60	All	All	All	2	1.44	1.05	0.24	0.12
Black grouper	All	All	Yes or maybe	All	2	1.44	1.05	0.24	0.12
Black grouper	All	All	Yes	All	2	1.44	1.05	0.24	0.12
Black grouper	All	>1000	All	All	2	1.44	1.05	0.24	0.12
**Black grouper**	**>60**	**>1000**	**Yes**	**Low**	**1**	**2.48**		**0.12**	
*Black grouper *	*>30 *	*All *	*Yes or maybe *	*Low to high *	*2 *	*1.44 *	*1.05 *	*0.24 *	*0.12 *

Mutton snapper	All	All	All	All	2	3.92	3.73	0.41	0.18
Mutton snapper	All	All	All	Low to moderate	1	7.64		0.58	
Mutton snapper	All	All	All	Low	0				
Mutton snapper	>30	All	All	All	1	7.64		0.58	
Mutton snapper	>60	All	All	All	1	7.64		0.58	
Mutton snapper	All	All	Yes or maybe	All	1	7.64		0.58	
Mutton snapper	All	All	Yes	All	1	7.64		0.58	
Mutton snapper	All	>1000	All	All	1	7.64		0.58	
**Mutton snapper**	**>60**	**>1000**	**Yes**	**Low**	**0**				
*Mutton snapper *	*>30 *	*All *	*Yes or maybe *	*Low to high *	*1 *	*7.64 *		*0.58 *	

Note: estimates in italics were published in Farmer and Ault (2011). Estimates in bold represent the most conservative filtering criteria explored.
